# Lung Cancer Risk after Exposure to Polycyclic Aromatic Hydrocarbons: A Review and Meta-Analysis

**DOI:** 10.1289/ehp.6895

**Published:** 2004-04-07

**Authors:** Ben Armstrong, Emma Hutchinson, John Unwin, Tony Fletcher

**Affiliations:** ^1^London School of Hygiene and Tropical Medicine, London, United Kingdom; ^2^Health and Safety Laboratory, Sheffield, United Kingdom

**Keywords:** cancer, lung, meta-analysis, PAH, polycyclics, review

## Abstract

Typical polycyclic aromatic hydrocarbon mixtures are established lung carcinogens, but the quantitative exposure–response relationship is less clear. To clarify this relationship we conducted a review and meta-analysis of published reports of occupational epidemiologic studies. Thirty-nine cohorts were included. The average estimated unit relative risk (URR) at 100 μg/m^3^ years benzo[*a*]pyrene was 1.20 [95% confidence interval (CI), 1.11–1.29] and was not sensitive to particular studies or analytic methods. However, the URR varied by industry. The estimated means in coke ovens, gasworks, and aluminum production works were similar (1.15–1.17). Average URRs in other industries were higher but imprecisely estimated, with those for asphalt (17.5; CI, 4.21–72.78) and chimney sweeps (16.2; CI, 1.64–160.7) significantly higher than the three above. There was no statistically significant variation of URRs within industry or in relation to study design (including whether adjusted for smoking), or source of exposure information. Limited information on total dust exposure did not suggest that dust exposure was an important confounder or modified the effect. These results provide a more secure basis for risk assessment than was previously available.

Airborne polycyclic aromatic hydrocarbons (PAHs), which are emitted when organic matter is burned, are ubiquitous in the occupational and general environment. It has long been known that several PAHs can produce cancers in experimental animals, and epidemiologic studies of exposed workers, especially in coke ovens and aluminum smelters, have shown clear excesses of lung cancer and highly suggestive excesses of bladder cancer [Boffetta et al. 1997; International Agency for Research on Cancer (IARC) 1984, 1985, 1987; Mastrangelo et al. 1996; Negri and La Vecchia 2001]. The animal experiments have included some using airborne exposure and have been mixtures and individual compounds, including particularly benzo[*a*]pyrene (BaP). Although the existence of a cancer risk is beyond reasonable doubt, considerable uncertainty exists as to the exposure–response relationship, and hence as to the risks posed at today’s levels in the workplace and general environment. Information on this relationship is clearly important for setting of occupational and environmental standards.

Estimating exposure–response relationships by extrapolation from animal studies is possible [Collins et al. 1991; U.S. Environmental Protection Agency (U.S. EPA) 1984], but the limitation of this approach, particularly species differences, makes sole reliance on it problematic. Data from a large cohort of coke oven workers in the United States, which has been followed since the 1960s (Costantino et al. 1995; Lloyd 1971), have been used to estimate risk per unit residential exposure [Nisbet and LaGoy 1992; World Health Organization (WHO) 1987]. However, many other studies provide information that has not yet been systematically used to quantitatively assess risk.

The fact that PAHs comprise a mixture, several components of which are animal carcinogens, adds to the complexity of the task. One issue is whether a single index of exposure, such as BaP or total benzene soluble matter (BSM) or cyclohexane soluble matter (CSM) is adequate to determine risk. If such an index is used, risk per unit exposure may differ between studies (and unstudied exposures) because of differences in the ratio of this index to the total carcinogenic potential of the mixture. It is possible that such variation, if present, can be adequately described by classifying exposures in broad categories (e.g., by source). However, this approach remains untested.

We conducted a review and meta-analysis that aimed to use all relevant published evidence from epidemiologic studies to obtain an estimate or estimates of the relationship of PAH exposure with lung and bladder cancer and to identify sources of variation in this relationship. Here we report the results for lung cancer.

## Methods

The methods summarized here are described at greater length in a technical report on this work (Armstrong et al. 2002).

### Literature Search

We sought all potentially informative peer-reviewed publications reporting epidemiologic studies on the occupational PAH–lung cancer exposure–response relationship. Specifically, we searched the following online electronic databases: MEDLINE (http://www.nlm.nih.gov/databases/databases_medline.html); EMBASE (http://www.embase.com/); OLDMEDLINE (http://www.nlm.nih.gov/databases/databases_oldmedline.html); NIOSHTIC-2 (http://www2.cdc.gov/nioshtic2/niosh2.htm); and CancerLit (http://www.cancer.gov/search/cancer_literature/). We searched publication dates 1958–February 2001) by text phrases and supplemented these publications with articles cited in the studies we obtained.

We excluded the following:

- Studies of workplaces where PAHs were considered unlikely to be the predominant lung or bladder carcinogen, to reduce potential for confounded results. Such workplaces included those in the rubber industry; those where primary exposure was from diesel exhaust; foundries; and steel works (because of co-exposure to silica), unless there were separate analyses specifically of coke oven workers.
- Studies for which it was not possible to quantify exposure to PAHs. Hospital- and population-based case–control and registry studies were excluded for this reason.
- Studies of occupational exposure other than by inhalation.
- Superceded publications. Where repeated follow-ups of the same workforce were reported in several articles, only the most recent was included.
- Biomarker studies because it was difficult to deduce relationships of exposure concentration to cancer incidence.
- Proportional cancer analyses.
- Articles not written in English.

After these exclusions, 34 articles remained, of which 5 reported two distinct cohorts for which results were presented separately. Thus, there were 39 cohorts. For each included cohort we systematically extracted general descriptive information, information on potential modifiers of risk associated with PAHs, and information from which we estimated unit relative risk (URR) increments (see next two subsections).

### Exposure Estimation

We distinguished studies according to author reports of the following:

- exposures to PAHs measured as BaP (10 cohorts)
- exposures to PAHs measured by a proxy that we could convert to BaP: benzene soluble matter (BSM), total PAHs, carbon black (6 cohorts)
- no measures of exposure (*n* = 23 cohorts)

For those studies with no exposure measures, we estimated exposure to PAHs for each workgroup for which cancer risk estimates were presented (e.g., for top-oven workers, side-oven workers). These exposure estimates were based on published exposure estimates in the same industries (IARC 1984; Lindstedt and Sollenberg 1982) and other published epidemiologic studies. Principle estimates thus derived are presented in Table 1.

#### Cumulative exposure.

We sought to relate cancer risk to mean cumulative exposures to BaP (duration × time-weighted mean concentration). Where risk by cumulative exposure was not published, it was derived as the product of mean estimated concentration of exposure in each group for which risk was reported and the mean duration of exposure in that group. In the absence of information on duration of exposure, 20 years was assumed, representing the average found in studies for which duration was reported.

#### Dust exposure.

At the inception of this study, we had not planned to seek information on potentially confounding exposures beyond those noted by the authors. However, interest has sharply increased recently in the hypothesis that inhaled dust carries a risk of lung cancer regardless of composition. We therefore sought to add information that we could find on dust exposure. Because few publications reported such estimates, we relied entirely on supplementary data and the judgment of the hygienists on the research team. We were aware that this would be a very rough assessment and chose a simple scale [low (< 1 mg/m^3^ total dust); moderate (1–5 mg/m^3^); high (5–10 mg/m^3^); very high (10–25 mg/m^3^)] and broad job groups or, in some cases, entire industries. Assessments are listed in the final column of Table 1. A list of references on which assessments were based is included in Appendix B4 of the full research report (Armstrong et al. 2002).

### Estimation of Unit Risks from Studies

We estimated relative risks (RRs) per 100 μg/m^3^ years cumulative BaP for each study, using a log-linear model, RR = exp(*bx*), where RR is relative risk, *x* is cumulative exposure in micrograms per cubic meter years and *b* is the slope of the exposure–response relationship. (For this model, RR = 1 if *x* = 0.) Thus, relative risk represents the risk of lung cancer at a specified exposure (*x*) relative to that at zero exposure. For example, RR = 1.30 at *x* = 100 μg/m^3^ years BaP exposure implies that at this exposure, lung cancer risk is 1.3 times that of an unexposed person—a 30% excess.

Slopes *b* were estimated by Poisson regression, using data from each study from published tables of risk [usually standard mortality ratios (SMRs) or internal RRs] by cumulative exposure, duration of exposure, or job group. For 13 cohorts with only one published SMR, these estimates depended on assuming that at zero exposure those cohorts would have experienced the same rates as those of the general population (allowing for age and calendar time). For the remaining 23 cohorts, rates at zero exposure were inferred from the cohort itself (i.e., exposure–response curves were not constrained to SMR = 1 at zero exposure). Standard errors were from a scale-overdispersion model, reflecting variation in observed deaths above the Poisson expected, and estimated jointly across studies.

Unit relative risks were defined as those predicted by the models at 100 μg/m^3^ years BaP [i.e., exp(100b)]. The exposure of 100 μg/m^3^ years BaP is close to the mean of the maximum exposures in included studies and corresponds to a concentration of 2.5 μg/m^3^ BaP over 40 years.

Many studies reported more than one contrast of risk in differently exposed subgroups, so several URRs could be estimated. For example, there may be tables of risk by duration of service (sometimes subdivided by job group) and by job group (e.g., coke oven top-worker, side-worker, and distillation products), as well as on overall SMR. In these instances we selected the following, in order of importance:

- internal comparisons (risk in groups of different exposure in the same study) over external (a single SMR)
- large contrasts of exposure across exposure groups
- mortality outcomes over morbidity
- confounder-controlled contrasts over uncontrolled (e.g., smoking-adjusted vs. unadjusted)
- estimates without latency or lag restrictions over those with such restrictions (to maximize comparability in primary analyses).

### Meta-Analysis and Meta-Regression

We sought to describe the distribution of URRs across studies and to identify determinants, allowing for sampling uncertainty of each estimate and additional random variation between studies (i.e., random effects) if present (Sutton 2000). We proceeded on the assumption that sampling variation of the logged URRs and any additional random variation were reasonably approximated by a normal distribution. Cochran’s test was used to determine significance of variation in URRs between studies. Meta-regression, using a log-linear random effects model with restricted maximum likelihood, was used to clarify patterns in URRs (e.g., a tendency for different URRs for each industry) and to identify whether such patterns could have occurred by chance (Sutton 2000).

## Results

### Study Characteristics

Characteristics of the 39 cohorts are shown in Table 2, and the frequencies of selected study characteristics are given in Table 3.

All were essentially cohort studies, but three used nested case–control samples, and one (Armstrong et al. 1994) used case-cohort sampling from within the cohort. For 13 of the cohorts, only single SMRs were reported; for the remainder there were risk comparisons (contrasts) across 2 or more exposure groups (maximum 7). Of these, the contrasts selected according to our criteria were by cumulative exposure (8), duration of exposure (12), and job group (6).

A remarkable feature was the large range of exposures. Table 2 lists the cumulative exposure in the highest exposure group in each study, which ranged across three orders of magnitude from 0.75 to 805 μg/m^3^ years BaP. This corresponds approximately to con-centrations in air of 0.04–40 μg/m^3^. This large range was the predominant reason for the large range in the precision with which the URR was estimated.

### Unit Relative Risks

Relative risks predicted at 100 μg/m^3^ years BaP from the log-linear model are shown to the right of the cohort characteristics in Table 2. They ranged from 0 to > 1,000. The precision with which these relative risks were estimated also varied substantially, with standard errors (log scale) ranging from 0.02 to >1,000. Most of the variation in precision was due to variation in the degree of exposure contrast in the studies. Many of the studies at the bottom of the table (power and carbon black industries) have low exposures. This limits the range of exposures being compared in the studies, which causes imprecision in estimated URRs, shown as wide confidence limits. (URRs are essentially regression coefficients—a narrow range in the *x* variable leaves uncertainty in the slope of the line.) Some variation in precision was also due to variation in size of cohort populations and duration of follow-up, which is reflected in the number of cases.

For cohorts without any exposure groups with mean higher than 100 μg/m^3^ BaP, the estimate of relative risk at this value (the URR) is an extrapolation. The extreme values of URRs found in such cohorts are thus theoretical. To give an indication of the actual relative risks found in the cohorts, we also show for each cohort (Table 2, last column) the relative risks for the group with the highest exposure in that cohort, as predicted by the (log-linear) model.

**Figure 1:**
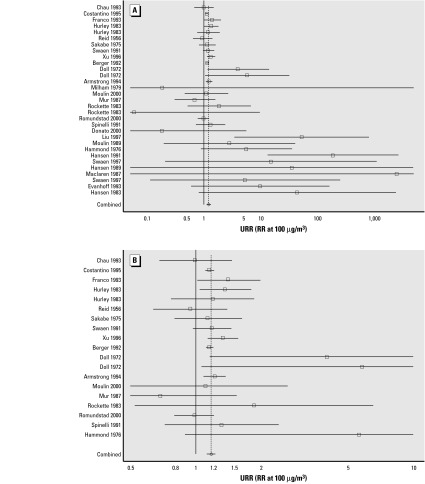
Estimated URRs (squares) and 95% (lines) for each cohort. Confidence intervals are truncated at the edges of the graph. Diamonds at the bottom of each show the overall mean and its confidence interval. The solid vertical line is at RR = 1, and the broken line is at the mean URR. (*A*) All studies with standard errors of log(URR) < 10. (*B*) Further restricted to studies with SEs < 1. Imprecise estimated URRs (SE > 10) are not graphed.

Twenty-eight (72%) of the URRs were > 1, with the lower confidence limit > 1 (*p* < 0.05) in 14 of these URRs. The mean (estimated by random effects meta-analysis), overall and in subgroups, is shown in Table 3. A graph of all results loses definition catastrophically in the more precise studies. Limiting the graph to studies with standard errors < 10 (Figure 1A) and 1 (Figure 1B) allows focus on the more precise and consequently influential cohorts.

The overall mean URR was 1.20 and significantly > 1 (*p* < 0.001). There was no one cohort dominating this estimate, and it was little changed on removal of the less precise cohorts. However, there was significant heterogeneity of URRs across cohorts (*p* < 0.001).

Meta-regression revealed that much of the heterogeneity was explained by variation in URRs across industries (*p* = 0.002), although coke ovens, gasworks, and aluminum smelters exposed to coal tar volatiles at similar levels had similar mean URRs. There was no significant heterogeneity of URRs within industry groups. We therefore examined variation in URRs according to other factors after allowing for the differences across industries by including industry in the meta-regression. After doing so, there was no difference more than could easily be explained by chance (*p* > 0.20) when studies were grouped according to source of exposure information, continent, whether the outcome of studies was mortality or morbidity, or exposure contrast (cumulative exposure, duration, etc). Neither did maximum exposure explain variation. The higher mean URR in the three nested case-control studies (*p* = 0.10) and that in the four smoking-adjusted studies (*p* = 0.05) are not independent. Both reflect high URRs in two case–control studies also adjusted for smoking.

### Publication Bias

There was little evidence that the URR was related to its standard error or to number of cases (*p* > 0.20), factors that might relate to publication. It is evident in Table 2 that although the very high URRs derive from the smaller studies with lower exposures, some of the extremely low estimated URRs do also. Further, neither Egger’s test nor Begg’s test (*p* > 0.20) gave evidence for publication bias (Sutton 2000). Applying a trim-and-fill analysis (designed to correct for publication bias, if any) made negligible difference to the mean.

### Dust

Because our information on dust exposure was for each cohort or sometimes for broad job group within studies (Table 1), we could not use conventional methods for controlling for confounding (stratification or inclusion of dust in multiple regression analyses). We adopted an ad hoc approach to use the data we had in order to shed what light we could on this issue:

We compared relative risks estimated at 100 μg/m^3^ years BaP in cohorts in which we had identified substantial dust exposure with those in which there was less. If generic dust were an important cause of lung cancer in these cohorts, one would expect greater apparent risks per unit PAH (BaP) where it was accompanied by dust. Results are shown at the bottom of Table 3. There was no significant association between estimated relative risk per unit PAH (BaP) exposure and dust exposure in the industry. This gives some reassurance that dust is not the predominant cause of the association seen in this cohort between PAH and lung cancer.

### Sensitivity Analysis

By investigating dependence of URRs on study characteristics (Table 3), we have already implicitly examined sensitivity of results to these characteristics (study design, smoking adjustment, exposure information, etc) and found little such sensitivity. Here we report investigations of sensitivity of our results to three statistical modeling assumptions.

First, we repeated analyses using the linear model (RR = 1 + *bx*). We found very similar rankings of URRs (Spearman’s correlation = 0.99). Fitted relative risks at the maximum exposure found in each plant were also similar. However, there was some variation in URRs of individual cohorts; those with lower exposures typically had lower URRs with the linear model, and those with higher exposures higher URRs. For example, the URR for Swaen’s 1997 study of asphalt workers was 15.23 with the exponential model but 3.13 with the linear model; the relative risk predicted at the actual mean exposure in this cohort of 10 μg/m^3^ years, however, was 1.31 for both models. Because methods are not available to rigorously allow for the highly non-regular sampling error in the linear estimates in meta-analyses, we view means and the assessment of heterogeneity of URRs estimated under this model cautiously. Nevertheless, it is reassuring that the mean estimated relative risk at 100 μg/m^3^ years BaP was similar (1.19 compared with 1.20, both highly significant). The patterns of variation of risk across industries were broadly similar, although with some important differences (e.g., means for coke, gas, aluminum, and other were 1.22, 2.25, 1.04, and 4.41, respectively, in linear model vs 1.17, 1.15,1.16, and 10.9, respectively, in log-linear model).

Second, we repeated analyses using alternative criteria for choice of contrast:

- Minimum standard error
- Minimum standard error but using internal comparisons instead of single SMRs whenever available.

In either case, the mean URR and the basic pattern of URRs between industries changed little, although estimates for individual studies changed, sometimes substantially.

Finally, we investigated dependence of our results on extrapolation of risks from very high exposures, by repeating analyses three times, excluding exposure groups with means more than 80 μg/m^3^ years BaP (40 years at 2 μg/m^3^), 40 μg/m^3^ years BaP (1 μg/m^3^), and 20 μg/m^3^ years BaP (0.5 μg/m^3^). For example, the large U.S. coke ovens study (Costantino et al. 1995) had seven groups with means 0.0, 14.8, 73.7, 162.4, 251.2, 339.9, and 805.4 but contributed only the first three groups to the first reestimated URR (means ≤ 80) and only the first two to the second and third reestimated URRs (means ≤ 40 and ≤ 20). Overall mean URRs and mean URRs for coke ovens, gasworks, and aluminum smelters are given in Table 4. The mean URR increases substantially on removal of higher exposure groups. This is partly explained by the greater weight given by URRs from industries with lower exposures, most of which have higher URRs. However, looking at the results for coke ovens, gasworks, and aluminum smelters only (right side of Table 4), we see that even within these industries restricting analyses to groups with lower cumulative exposures led to higher mean URRs, suggestive of an exposure–response curve steeper at lower exposures than at higher exposures. However, for all these analyses except those excluding all exposures above 20 μg/m^3^ years BaP, which was imprecise, there was significant heterogeneity between studies. These results should therefore be interpreted with caution.

## Discussion

That our meta-analysis supports the conclusions of previous reviews that lung cancer is associated with PAH exposure is reassuring but not surprising. Our attention to quantification of this relationship in a comprehensive review is novel. Although other reviews have cited unit risk estimates from single studies, and one (Gibbs 1997) calculated such estimates from eight studies, no meta-analyses of unit risk estimates have been published.

Our results for coke ovens, gasworks, and aluminum production are relatively well supported by evidence from multiple studies, although biases should be considered. Our findings of higher URRs for other industries are more tentative. In the following sections, we discuss biases and possible explanations for patterns of variation in URRs.

### Possible Biases

Each study included in this meta-analysis is subject to the usual range of potential biases in epidemiologic studies, in particular, confounding and information bias (exposure error).

Our first concern is potential confounding by smoking, which was uncontrolled in most studies. However, for two reasons, this seems unlikely to have caused major bias: *a*) Although only four studies controlled for smoking, two were large studies with substantial exposure allowing precise estimates of URRs. The mean URR in smoking-adjusted studies was statistically compatible with but somewhat higher than that for the studies uncontrolled for smoking and was statistically significant. *b*) Several methodological articles (Axelson and Steenland 1988; Blair et al. 1988; Siemiatycki et al. 1988) have explored mathematically the potential for confounding by smoking. One common conclusion was that because comparisons are generally between groups with only moderately differing smoking habits (particularly different groups of manual workers, as in most studies in this analysis), substantial confounding is unlikely.

Confounding by other occupational exposure is also possible, but we limited that potential by excluding cohorts in which PAHs were judged unlikely to be the predominant carcinogen. We did not exclude subjects exposed to high levels of total dust, however, because the hypothesis that dust may cause lung cancer regardless of composition has gained credence only recently (Pope et al. 2002) and because dust is a universal co-exposure of PAHs. The analysis that we conducted addressing the possibility of confounding by dust gave no support to the hypothesis that dust plays a major confounding role. Other ad hoc investigations of confounding potential, in particular noting the absence of lung cancer excess in prebake aluminum workers (exposed to dust but little PAH), came to similar conclusions (Armstrong et al. 2002). However, none of our analyses could rule out confounding completely because of the limited information on total dust exposure available to us and the lack of control for this exposure in the published studies. Further evaluation will be possible when assessments of the dust hypothesis are carried out, which was not possible in this study.

Exposure is likely to have been inaccurately estimated in many studies, in particular those for which no exposure data were published in the report of the epidemiologic study itself, so we made estimates. Random exposure error tends to bias exposure–response slopes toward the null value (Armstrong 1998). However, if our estimates were systematically too high or too low, exposure response slopes would be underestimated or overestimated, respectively. We included estimates if we believed them to be within 4 times the true exposure, so considerable margin for uncertainty remains. It is somewhat reassuring that the mean URR in those studies for which we estimated exposure was not much different from the mean URR in those studies with author-provided exposure information (Table 3). However, errors in exposure estimation might explain particularly high or low URRs in specific studies or industries. In those industries (tar distillation, chimney sweeping, power) with no studies reporting investigators’ own exposure estimates, interpretation should be particularly cautious.

Finally, could bias be introduced by selection of cohorts or contrasts for inclusion? Our sensitivity analyses suggest no strong sensitivity, and standard tests for publication bias were negative. However, the overall mean URR is strongly influenced by the predominance of coke ovens and aluminum smelters in the sample.

### Explanations for Variation in Unit Relative Risks

Unit relative risks may vary between industries and cohorts for three reasons: *a*) chance, *b*) biases, or *c*) because risk per unit BaP really varies. We established that variation in URRs between industries cannot be explained by chance (particularly coke ovens and aluminum production vs. asphalt and chimney sweeping), but variation within industry can be. Therefore, it seems sensible to focus attention on explaining variation between industries. We discussed biases and confounding in the preceding section. Biases, in particular from inevitably inaccurate exposure estimation, could explain some variation. Confounding by other occupational exposures, perhaps dust, could also play a part, although we found no evidence for this.

Two reasons might account for true variation in URRs. *a*) A factor that modifies the effect is present to varying degrees in different industries. An example is smoking. Even if different PAH exposure groups in each cohort smoke to the same extent (so there is no confounding), a heavily smoking cohort might exhibit greater or lesser effect on relative risk per unit occupational PAH than a lightly smoking cohort. Unfortunately, we did not have the information to address this aspect. Generally, we can assume that our cohorts were mixed smokers and nonsmokers, so the exposure–response relationships are most likely to predict risk well in similarly mixed groups. Other occupational exposures might also modify the effect per unit PAH by promoting or inhibiting the action of PAHs. Such a hypothesis is too general to evaluate without making it more specific. Finally, cumulative exposure may not be the right metric. If another metric (e.g., early adult exposure, lagged exposure, or another time-weighted exposure) were the relevant one, modification would arise if the time pattern of exposure differed across cohorts. Information on timing of exposure was insufficient for us to evaluate such hypotheses, but in any case we expect that timing of exposure would be too similar across cohorts for informative results to emerge. A few studies reported risk by lagged cumulative exposure, but these generally differed little from tables of risk by overall cumulative exposure. *b*) The carcinogenic potency of the PAH mixture varies across industries. As we noted earlier, many PAHs aside from BaP are carcinogenic in animals (IARC 1985). BaP is used as an indicator of the total risk, not because it is the sole causal agent but because at least in some industries it correlates well with other agents (Expert Panel on Air Quality Standards 1999). To the extent that PAH mixtures in different industries have different relative concentrations of the various carcinogenic PAHs (their profiles), this could thus explain differences in risk per unit BaP. Krewski et al. (1989) have proposed an approach that derives a risk metric by combining information on PAH profiles with information on relative carcinogenic potency from animal studies. To apply that approach to this meta-analysis, however, would require estimates of PAH profiles for each study or at least each industry. We did not have such information, which is not readily available, for this study. However, PAH profiles are slowly being ascertained and some are published (Appendix B3, Armstrong et al. 2002), so this approach could probably be applied in the future.

Some specific studies with URRs quite different from the mean for their industry deserve specific mention:

- The two cohorts of gasworks worker studies (Doll et al. 1972) have high URRs. The estimate of exposure for retort workers in these plants was 3 μg/m^3^ BaP and was heavily influenced by measurements reported in 1965 from mask samples. These measurements may have been underestimates.
- The very high URR estimated from one study of carbon anode plant workers (Liu et al. 1997) was based on exposure estimates reported by Liu for just one of seven plants, which may not have been representative.
- The low and precisely estimated URR from the study of several Norwegian aluminum production plants (Romundstad et al. 1998) has no obvious explanation. Exposure estimation was based on substantial hygiene data for most plants. The nonsignificance of the test for heterogeneity in URRs among studies of aluminum production workers indicates that the absence of excess risk in this study could have been due to chance, but the result remains noteworthy.

### Comparisons with Other Unit Risk Assessments

The most directly comparable study estimated lifetime risks of lung cancer per 100,000 men from 50 years of continuous exposure to 1 ng/m^3^ BaP [unit lifetime risk (ULR)] from nine studies, using a linear no-threshold model (Gibbs 1997). Eight of the nine studies were occupational (four from coke ovens, two gasworks, one aluminum production, and one asphalt), and they or their updates were included in our analysis. The ninth was a study of domestic exposure to smoky coal in China. To translate Gibbs’ risk lifetime estimates from continuous exposure to relative risk estimates from occupational exposure (per 100 μg/m^3^ BaP), we used the conversion factors used by Gibbs to do the reverse:


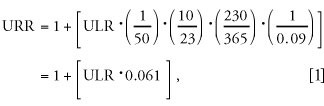


where ULR is the lifetime risk per nanogram per cubic meter continuous (23 m^3^/day vs. 10 occupational) exposure (365 days vs. 230 occupational—our assumption) over 50 years, assuming a 9% baseline lifetime risk. Gibbs’ finding of ULR 0.3, 4.2, 4.4, 5.8, 6.6, 7.2, 7.8, and 9.5 translates to URRs 1.02, 1.26, 1.27, 1.35, 1.40, 1.44, 1.48, and 1.58, which are somewhat higher on average than the estimates for the same studies in this analysis but not grossly different.

It is also possible to compare our meta-analytic estimates with those published from the U.S. coke oven cohort (latest update reported by Costantino 1995):

- The U.S. EPA (1984), cited by WHO (1987) estimated a lifetime risk from continuous exposure per nanogram per cubic meter BaP of 8.7/100,000 using the linearized multistage model. Following the translation we used for the Gibbs study, this corresponds to a URR (relative risk from 100 μg/m^3^ years BaP) of about 1.53.
- Moolgavkar et al. (1998) estimated a unit absolute risk from continuous 1 μg/m^3^ BSM of 15/100,000 using the two-stage clonal expansion model. Roughly re-expressing this using the Gibbs study translation gives a URR = 1.13.

The second of these alternative estimates of URR is very similar to our estimate from Costantino (1995) using the exponential model (Table 2; URR = 1.15).

A review of 10 studies with risk estimates published for two or more exposure groups (Mastrangelo et al. 1996) emphasized the unit risk estimate published in one aluminum smelter study (Armstrong et al. 1994). The unit risk estimate cited by Mastrangelo for the Armstrong study used the linear model and was somewhat higher (1.39 translated into the units we have used) than the log-linear URR for this study (1.22) that we used in our meta-analysis.

Our findings of a larger URR in the asphalt industry than in coke ovens or aluminum smelters was tentative. Recent publication of a very large European study of mortality in the asphalt industry (Boffetta and Burstyn 2003) will add important information on this question. The study was not published in time for formal inclusion in this meta-analysis. It found an association of lung cancer with exposure to bitumen fumes in some but not other analyses. Estimates of exposure to PAHs as BaP were made, allowing as far as possible for knowledge of the extent to which coal tar was used as an additive, time trends in exposure levels, and type of asphalt paving. In the asphalt industry PAH exposure originates from bitumen, coal tar (now banned in Western Europe), and diesel exhaust. Contribution of diesel exhaust to PAH exposure was not incorporated into quantitative PAH exposure metric because available data did not permit the investigators to identify groups of asphalt pavers within the cohort with different diesel exhaust exposure. The technical report of this study (Boffetta et al. 2001) includes a table (8.9.4) of lung cancer rate ratios in relation to cumulative exposure to PAHs (as BaP). From this table, it was possible to estimate a URR in the method that was standard for our meta-analysis. The estimate [44.9; 95% confidence interval (CI), 25.0–64.8] is similar to the that of other asphalt worker studies included in this review, adding support to the hypothesis that risk per unit BaP is higher in this industry than in coke ovens or aluminum production. However, analysis of risk by quantitative estimate of PAH exposure was possible only for workers employed in paving (including mastic paving). It may be that other groups in the study (e.g., roofers) showed different patterns.

### Interpretation for Risk Assessment

We have used a benchmark of 100 μg/m^3^ years exposure to provide a scale for presenting the URR, but risk predictions at other exposures (*x*) can be made using the formula


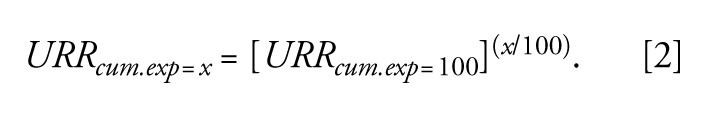


For example, relative risk consequent on exposure to 1 μg/m^3^ for 40 years (40 μg/m^3^ years) according to the mean estimate for coke ovens is 1.17^(40/100)^ = 1.06. (At these moderate to low relative risks, log-linear interpolation is close to linear interpolation.) Risk estimates calculated this way for a range of URRs and exposure concentrations are given in Table 5.

#### Overall or industry-specific means?

The URRs overall had significant and substantial heterogeneity. There was evidence that risk per unit BaP varied across cohorts. The mean in the presence of this heterogeneity is a rather artificial one, reflecting those industries and cohorts that happen to have been studied. Within industries there was no significant heterogeneity, so that the industry-specific means could be interpreted as representative of each industry. These considerations favor use of industry-specific means. Means for coke ovens, gas works, and aluminum production are consistent and relatively precisely estimated. The combined mean URR for these industries was 1.17 (95% CI, 1.12–1.22) and might reasonably be used for all these industries. However, means for other industries are imprecise. Risk assessment for these industries will inevitably be uncertain, whether the imprecise industry-specific mean or the overall mean was used.

#### Model choice.

Risk assessment depends on the form of the model, in particular for extrapolation of risk to exposure ranges far from those observed. We adopted the log-linear model because the linear model is not amenable to rigorous statistical evaluation; estimates and confidence intervals for means, and p-values for heterogeneity are unreliable. However, evidence suggests (Appendix C, Armstrong et al. 2002) that the linear model fits the data and arguments on mechanism better than the log-linear model. That the overall mean and broad pattern of URRs under the linear and log-linear models were similar is reassuring, but having model choice forced by statistical tractability is not ideal. The development of methods to allow better meta-analysis of linear relative risk models would be useful.

Apart from the log-linear and linear models, models with very different assumptions about increments at low exposures, such as threshold models, could predict very different risks at these levels. However, information was insufficient to fit these or other more elaborate models (e.g., two-stage, multistage) with the information published. In particular, lack of information precluded our investigating dependence of risk on timing or exposure beyond the cumulative exposure model, for example, risk eventually declining after exposure. The sensitivity analysis (Table 4) investigating dependence of results on high exposures was suggestive of an exposure–response curve steeper at lower exposure than at higher exposure.

#### Attributable burden of disease.

The number of cancers caused by occupational exposure to PAHs depends on three factors beyond the exposure–response relationship: *a)* the number of persons exposed; *b)* the levels at which they are exposed, and *c)* the background rate of lung cancer on which relative risks will act. As an example, we have made an estimate of cases that would be caused in U.K. coke oven workers by PAH exposures continuing at current levels, ignoring probably higher past exposures. There are currently about a thousand coke oven workers in the United Kingdom, with mean exposure about 1.5 μg/m^3^ BaP (Unwin J, personal communication). General population lifetime risk of lung cancer in U.K. males, using 1997 rates, is 8% (Office for National Statistics 2000). Using the mean URR of 1.17 for coke ovens, 1 year of exposure will therefore lead eventually to a lifetime excess risk of 0.08 × (1.17^(1.5/100)^–1) = 1.9 × 10^–4^, which among 1,000 workers will lead to 0.2 cases. Forty years of such exposure would lead to 40 × 0.2 = 8 cases.

#### Assessing risk in the general environment.

Included cohorts were all occupationally exposed, and our study was aimed primarily at informing risk assessment in an occupational setting. However, given the limited number and informativeness of direct studies of risks from PAHs in the general ambient exposure, these data also provide a possible basis for estimating these risks. A full discussion is beyond the scope of this article, but we note that for this purpose our (occupational) ULRs would have to be converted to apply to continuous (24-hr, 365-day) exposure, such as with the assumptions of Gibbs discussed above.

#### Uncertainty.

We have acknowledged many sources of uncertainty in risk estimates made from a summary URR. Many such sources, notably model choice and exposure uncertainty, are not incorporated in the confidence intervals, which should be regarded as lower bounds of uncertainty.

### Methodological Lessons

Compared with their widespread use in clinical trials, meta-analyses are relatively new to occupational epidemiology, and even more rare in investigations of exposure–response relationships. In entering this poorly charted territory, this study presented several methodological challenges for which we found reasonable but ad hoc solutions. It might be useful to future similar meta-analyses for us to draw attention to the principle issues:

- We needed to choose one contrast from each study from which to estimate an exposure–response relationship. To be objective, we selected a simple choice algorithm and explored sensitivity of results to it, but it may be that this procedure could be improved.
- We needed to estimate mean exposure in upper-exposure groups for which only a lower limit was published.
- *A priori* considerations and data in the meta-analysis studies suggested use of linear rather than log-linear models, but estimates of URRs from linear models proved intractable in meta-analysis, so we worked with log-linear models. It would be preferable not to have to compromise. One possibility would be to apply a random effects linear relative risk model to semiaggregated data (see below), but to our knowledge, such models have not been discussed in the statistical literature, nor can they be fitted with standard software.
- We proceeded in this meta-analysis to estimate first a single effect measure (URR) from each study, then analyze these measures using standard meta-analytic methods. However, it appears to us that once semiaggregated data have been assembled for cases, exposures, and relative risks in each exposure group in each study (Appendix E, Armstrong et al. 2002), it would be possible to use methods developed more generally for hierarchical data (multilevel models).

## Conclusion

Considerable independent data are now available that allow us to conclude that occupational exposure to PAHs by inhalation is associated with a risk of lung cancer. For exposures in the coke ovens, gasworks, and aluminum industries, the risk can be estimated and is equivalent to a relative risk of 1.06 for a working lifetime at 1 μg/m^3^ exposure to BaP. Exposures in other industries with PAH exposure, in particular carbon anode plants, asphalt use, and tar distilleries, suggest higher risks at equivalent BaP exposure, but the risk estimates are much less precise.

## Figures and Tables

**Table 1:** Main supplementary exposure estimates (BaP; total dust).

Industry	Job group	BaP (μg/m^3^)	Dust*^a^*
Coke ovens	Top	20	H
	Side	10	M
	Other	0.5	M
	Typical plant mean	10	H/M
Coal gas production	Retorts	3	M
	By-products	0.5	L
	Typical plant		M/L
Aluminum smelting	Soderberg potroom	15	VH
	Prebake potroom	0.05	H
	Carbon plant	2	H
	Typical plant mean: Soderberg	3	H
	Typical plant mean: Prebake	0.5	H/M
Carbon anode plants	—	1	H
Asphalt	—	0.5	M
Tar distillation	—	0.5	M
Chimney sweep	—	1	VH
Thermoelectric power	—	0.05	L
Carbon black	—	0.05	H

*^a^*Dust classification: L, low (< 1 mg/m^3^); M, moderate (1–5 mg/m^3^); H, high (5–10 mg/m^3^); VH, very high (10–25 mg/m^3^).

**Table 2:** Cohort characteristics and URR estimates.

	Cohort characteristics										URR		RR
First author and year	Industry*^a^*	Country	Design*^b^*	Author exposure*^c^*	Contrast*^d^*	Outcome*^e^*	Smoking adjustment	Cases (*n*)	Exposure (*n*)	Maximum exposure*^f^*	Estimate (95% CI)	SE*^g^*	Maximum exposure
Bye 1998	Coke	Norway	Cohort	Proxy	cum.exp.	Morb	No	7	4	10.0	> 1,000 (0.01 to > 1,000)	11.43	6.10
Chau 1993	Coke	France	Cohort	None	jobgroup	Mort	Yes	24	5	294.4	1.00 (0.68–1.46)	0.20	0.99
Costantino 1995	Coke	USA	Cohort	Proxy	cum.exp.	Mort	No	458	7	805.4	1.15 (1.10–1.21)	0.02	3.18
Franco 1993	Coke	Italy	Cohort	None	singleSMR	Mort	No	19	1	186.0	1.41 (1.01–1.97)	0.17	1.90
Hurley 1983	Coke	U.K.	Cohort	Proxy	cum.exp.	Mort	No	182	4	252.9	1.36 (1.04–1.79)	0.14	2.19
Hurley 1983	Coke	U.K.	Cohort	Proxy	cum.exp.	Mort	No	59	4	262.9	1.19 (0.77–1.85)	0.22	1.60
Reid 1956	Coke	U.K.	Cohort	None	jobgroup	Mort	No	21	3	400.0	0.94 (0.64–1.39)	0.20	0.79
Sakabe 1975	Coke	Japan	Cohort	None	singleSMR	Mort	No	15	1	200.0	1.13 (0.80–1.60)	0.18	1.28
Swaen 1991	Coke	Holland	Cohort	None	jobgroup	Mort	No	273	3	200.0	1.19 (0.97–1.45)	0.10	1.41
Xu 1996	Coke	China	Nested	BaP	duration	Morb	Yes	194	3	453.8	1.33 (1.14–1.56)	0.08	3.65
Berger 1992	Gas	Germany	Cohort	BaP	singleSMR	Mort	No	78	1	747.6	1.15 (1.11–1.20)	0.02	2.88
Doll 1972	Gas	U.K.	Cohort	None	jobgroup	Mort	No	79	3	60.0	4.01 (1.16–13.87)	0.63	2.30
Doll 1972	Gas	U.K.	Cohort	None	jobgroup	Mort	No	110	2	60.0	5.82 (1.06–32.00)	0.87	2.88
Gustavsson 1990	Gas	Sweden	Cohort	BaP	singleSMR	Mort	No	0	1	28.7	0.00 (0.00–66.56)	1,450	0.00
Armstrong 1994	Alum	Canada	Ca-coh	BaP	cum.exp.	Mort	Yes	338	5	413.1	1.22 (1.09–1.37)	0.06	2.30
Milham 1979	Alum	USA	Cohort	None	duration	Mort	No	35	6	99.2	0.19 (0.00 to > 1,000)	6.15	0.19
Moulin 2000	Alum	France	Cohort	None	duration	Mort	No	19	5	200.0	1.11 (0.46–2.66)	0.45	1.23
Mur 1987	Alum	France	Cohort	None	duration	Mort	No	17	3	248.2	0.69 (0.31–1.54)	0.41	0.40
Rockette 1983	Alum	USA	Cohort	None	duration	Mort	No	64	5	116.1	1.85 (0.53–6.53)	0.64	2.05
Rockette 1983	Alum	USA	Cohort	None	duration	Mort	No	133	5	15.4	0.06 (0.00–9.58)	2.59	0.65
Romundstad 2000	Alum	Norway	Cohort	BaP	cum.exp.	Morb	No	189	4	222.4	0.99 (0.79–1.22)	0.11	0.97
Spinelli 1991	Alum	Canada	Cohort	Proxy	cum.exp.	Morb	No	37	5	251.1	1.31 (0.72–2.39)	0.30	1.99
Donato 2000	Carbon	Italy	Cohort	None	duration	Mort	No	34	3	36.4	0.18 (0.01–5.61)	1.75	0.54
Liu 1997	Carbon	China	Cohort	BaP	jobgroup	Mort	No	50	4	17.3	53.07 (3.44–819)	1.40	1.99
Moulin 1989	Carbon	France	Nested	BaP	duration	Morb	Yes	7	4	94.9	2.82 (0.20–40.59)	1.36	2.67
Moulin 1989	Carbon	France	Nested	BaP	duration	Mort	No	13	4	5.8	0.00 (0.00 to > 1,000)	24.21	0.41
Hammond 1976	Asphalt	USA	Cohort	BaP	duration	Mort	No	121	4	66.8	5.63 (0.89–35.53)	0.94	3.17
Hansen 1991	Asphalt	Denmark	Cohort	BaP	singleSMR	Mort	No	25	1	20.3	189.59 (13.5 to > 1,000)	1.35	2.90
Swaen 1997	Asphalt	Holland	Cohort	None	singleSMR	Mort	No	39	1	10.0	15.23 (0.21 to > 1,000)	2.19	1.31
Hansen 1989	Tar	Denmark	Cohort	None	singleSMR	Mort	No	16	1	10.0	35.76 (0.04 to > 1,000)	3.42	1.43
Maclaren 1987	Tar	U.K.	Cohort	None	singleSMR	Mort	No	12	1	6.0	> 1,000 (0.01 to > 1,000)	6.58	1.60
Swaen 1997	Tar	Holland	Cohort	None	singleSMR	Mort	No	48	1	10.0	5.32 (0.11–89.4)	1.97	1.18
Evanhoff 1993	Chimney	Sweden	Cohort	None	duration	Mort	No	53	4	40.0	9.88 (0.60–162)	1.43	2.50
Hansen 1983	Chimney	Denmark	Cohort	None	singleSMR	Mort	No	5	1	30.0	44.63 (0.82 to > 1,000)	2.04	3.13
Cammarano 1986	Power	Italy	Cohort	None	singleSMR	Mort	No	5	1	1.0	> 1,000 (0.00 to > 1,000)	61.16	1.77
Forastiere 1989	Power	Italy	Cohort	None	duration	Mort	No	8	3	1.5	0.02 (0.00 to > 1,000)	110.37	0.94
Petrelli 1989	Power	Italy	Cohort	None	singleSMR	Mort	No	6	1	1.0	> 1,000 (0.00 to > 1,000)	55.83	1.36
Robertson 1996	C_black	USA	Cohort	None	singleSMR	Mort	No	34	1	1.0	0.00 (0.00 to > 1,000)	23.45	0.84
Sorahan 2001	C_black	U.K.	Cohort	Proxy	cum.exp.	Mort	No	64	4	0.8	> 1,000 (0.00 to > 1,000)	58.15	1.48

**a**Industry: Alum, aluminum smelter; Carbon, carbon anode plant; Tar, tar distillery; Chimney, chimney sweep; Power, thermoelectric power plant; C_black, carbon black.

**b**Design: Nested, nested case–control; Ca-coh, case–cohort.

**c**Author exposure: information provided by the authors on exposure to BaP.

**d**Contrast: Basis of risk comparison from which URR was estimated. cum.exp., cumulative exposure.

**e**Outcome: Morb, morbidity; Mort, mortality.

**f**Exposure in micrograms per cubic meter years BaP in the highest exposure group.

**g**Standard error of URR (log scale).

**Table 3:** Distribution and determinants of URRs.

Group	Studies (*n*)	Mean URR*^a^* (95% CI)	Significance tests*^b^*
All cohorts	39	1.20 (1.11–1.29)	*p*(het) = 0.007
Excluding less precise URR estimates			
Restricted to URRs with SE < 10	31	1.20 (1.11–1.30)	*p*(het) = 0.002
Restricted to URRs with SE < 1	19	1.18 (1.12–1.23)	*p*(het) = 0.19
By industry			*p* = 0.002
Coke ovens	10	1.17 (1.12–1.22)	
Gasworks	4	1.15 (1.11–1.20)	
Aluminum	8	1.16 (1.05–1.28)	
(above three combined)	22	1.17 (1.12–1.22)	*p*(het) > 0.20]
Carbon	4	4.30 (0.81–22.79)	
Asphalt	3	17.50 (4.21–72.78)	
Tar distillery	3	12.28 (0.48–314.4)	
Chimney sweep	2	16.24 (1.64–160.7)	
Power	3	> 1,000 (0 to > 1,000)	
Carbon black	2	0 (0 to > 1,000)	
By exposure information from authors			*p* > 0.20
BaP	10	1.29 (1.11–1.49)	
Proxy	6	1.16 (1.11–1.21)	
None	23	1.17 (1.03–1.33)	
By contrast			*p* > 0.20
Cumulative exposure	8	1.16 (1.11–1.22)	
Duration	12	1.27 (1.10–1.48)	
Job group	6	1.16 (0.99–1.36)	
Single SMR	13	1.20 (0.95–1.51)	
By study design			*p* = 0.10
Cohort	36	1.16 (1.11–1.21)	
Nested case–control	3	1.33 (1.14–1.55)	
By smoking adjustment			*p* = 0.05
No	35	1.16 (1.11–1.21)	
Yes	4	1.31 (1.16–1.48)	
By continent			*p* > 0.20
Asia	3	1.30 (1.13–1.50)	
Europe	28	1.13 (1.02–1.26)	
North America	8	1.16 (1.11–1.22)	
By outcome			*p* > 0.20
Mortality	34	1.17 (1.12–1.22)	
Morbidity	5	1.21 (1.06–1.38)	
By dust exposure for industry			*p* = 0.12
Low	3	> 1,000 (0 to > 1,000)	
Moderate	10	1.16 (1.11–1.21)	
High	24	1.17 (1.13–1.22)	
Very high	2	16.24 (1.64–14.8)	

**a**RR at 100 μg/m^3^ BaP years. Adjusted for differences across industries by including industry indicator in a meta-regression. Means are scaled to show fitted values for coke ovens, although ratios would apply to any industry.

**b**Generally, the Wald test for significance of variation in mean URRs across the groups indicated was used; “*p*(het)” indicates the test for heterogeneity across all studies.

**Table 4:** Investigating the dependence of mean URR on high exposures.

	All studies			Coke, gas, aluminum		
Exclusions	*n**^a^*	URR (95% CI)	*p*(het)*^b^*	*n**^a^*	URR (95% CI)	*p*(het)*^b^*
No exclusions	39	1.20 (1.11–1.29)	< 0.001	22	1.17 (1.12–1.22)	0.20
> 80 μg/m^3^	34	3.46 (2.03–5.90)	< 0.001	17	1.88 (1.22–2.91)	0.02
> 40 μg/m^3^	30	6.49 (1.99–21.12)	< 0.001	14	2.42 (0.56–10.40)	0.01
> 20 μg/m^3^	21	4.54 (1.26–16.30)	> 0.20	7	1.87 (0.24–14.22)	> 0.20

*^a^*Remaining number of studies from which URRs could be estimated.

*^b^*Test for heterogeneity between URRs.

**Table 5:** Relative risks for contracting cancer estimated to follow from 40 years of occupational exposure.

	Exposure to BaP in μg/m^3^ for a working life of 40 years			
URR used for circulation	0.1	0.2	0.5	1
Overall mean URR (1.20)	1.007	1.015	1.035	1.076
Mean URR for coke ovens, aluminum smelters, and gasworks (1.16)	1.006	1.012	1.030	1.061
Mean URR for asphalt (17.5)	1.12	1.26	1.78	3.14
